# Uncovering Barriers to the Diagnosis and Treatment of Metastatic Melanoma: A Case Report

**DOI:** 10.7759/cureus.75716

**Published:** 2024-12-14

**Authors:** Samone Alexander, Marina M Georgies, Yoelbis Alcolea Tamayo, Maria Abeysekera

**Affiliations:** 1 College of Medicine, California Northstate University College of Medicine, Elk Grove, USA; 2 Department of Family Medicine, Saint Agnes Medical Center, Fresno, USA

**Keywords:** foot ulcers, insurance barriers, language barriers, metastatic melanoma, socioeconomic barriers

## Abstract

Melanoma is a malignant disorder of the skin that originates from melanocytes. It is the most aggressive of the skin malignancies. This case study presents a unique case of a 52-year-old male gardener with melanoma on the plantar side of his foot, which progressed to a large ulcer. Delayed care, attributed to insurance mistakes and language barriers, allowed for advancing the patient's condition, highlighting the detrimental impact of healthcare barriers on patient outcomes. The case emphasizes the urgent need for simplified and efficient healthcare processes and access to timely intervention. Addressing systemic disparities and implementing culturally competent care practices are necessary to provide equitable access to healthcare, reduce disease progression, and improve patient outcomes.

## Introduction

​​​Melanoma can present a challenge in clinical practice due to its potential for aggressive behavior and metastasis to multiple sites such as the lungs, liver, brain, and bone [1]. It typically manifests as a pigmented lesion on the skin, evolving either from pre-existing nevi or as de novo formations [2]. Ultraviolet radiation is the most common environmental risk factor for melanoma; therefore, it most often affects sun-exposed areas. However, melanoma can arise anywhere on the body, including acral sites such as the plantar surface of the foot [3]. Acral melanomas often present diagnostic challenges and are associated with delayed diagnoses and poorer prognoses [4,5]. The diagnosis and treatment of melanoma are also influenced by the socioeconomic status of the patient. In a retrospective study by Amini et al., it was found that metastatic melanoma rates were 10.3% higher among uninsured patients compared to those with non-Medicaid insurance [6]. Barriers to medical care are associated with adverse effects and poor patient outcomes [7].

This report reviews the case of a 52-year-old male gardener who initially presented with a lesion on the plantar side of his foot that progressed into a large ulcer and was later diagnosed as melanoma. Delayed treatment resulted from a complex intersection of barriers in healthcare access, including insurance errors, cultural beliefs, and language barriers hindering effective communication with healthcare providers. These factors exacerbated the patient's condition, highlighting the detrimental effects of such barriers. Therefore, addressing systemic disparities and implementing culturally competent care are essential to ensure equitable access to healthcare and consequently improve patient outcomes.

## Case presentation

On December 6, 2022, a 52-year-old male patient presented to the clinic with a chief complaint of an ulcer on the plantar aspect of his left foot, near the base of the first and second metatarsals. His medical history included type 2 diabetes mellitus and hyperlipidemia, and his family history was negative for cancer. He initially noticed the lesion approximately five months earlier as a small black spot. During a previous emergency room visit, he underwent a foot X-ray, which was negative for osteomyelitis and was discharged with antibiotics. The lesion progressively developed into an 8-cm deep necrotic ulcer with indurated borders. The surrounding tissue was erythematous, swollen, and warm, with no purulent discharge. The patient had intact distal sensation and no peripheral neuropathy. He also presented with lumps on his left arm, neck, and right abdomen, which he had noticed one to two months earlier. At this time, the immediate management plan included an MRI, podiatry referrals, wound care referrals, wound dressing, and crutches.

At a follow-up visit on December 13, 2023, the ulcer remained unchanged, with decreased foot swelling. On physical examination, the clinician observed a 3-cm lump on the left antecubital fossa, 5-mm lumps on his bilateral neck, and an 8-mm lump on his right abdomen; all were circular, rubbery, and movable. At this visit, insurance issues became evident: MRI, podiatry referral, and wound care referral were denied due to the patient’s dual insurance status. The treatment plan could not be initiated until the patient was registered under a single primary insurance that was consistent with the clinic's registration file. The patient and his sister (who was also his translator) were previously unaware of his dual insurance status and were provided instructions on how to resolve the issue. The patient was scheduled for a follow-up visit in one month and was advised to seek an earlier appointment if possible.

On February 2, 2023, the patient returned for follow-up after resolving his insurance issues. He presented with new, nontender, mobile nodules on his left arm, right neck, left foot, chest, and back, which he reported had appeared two months earlier and were growing. The previous treatment plan was expanded to include ultrasounds of these nodules as well as bloodwork.

**Figure 1:**
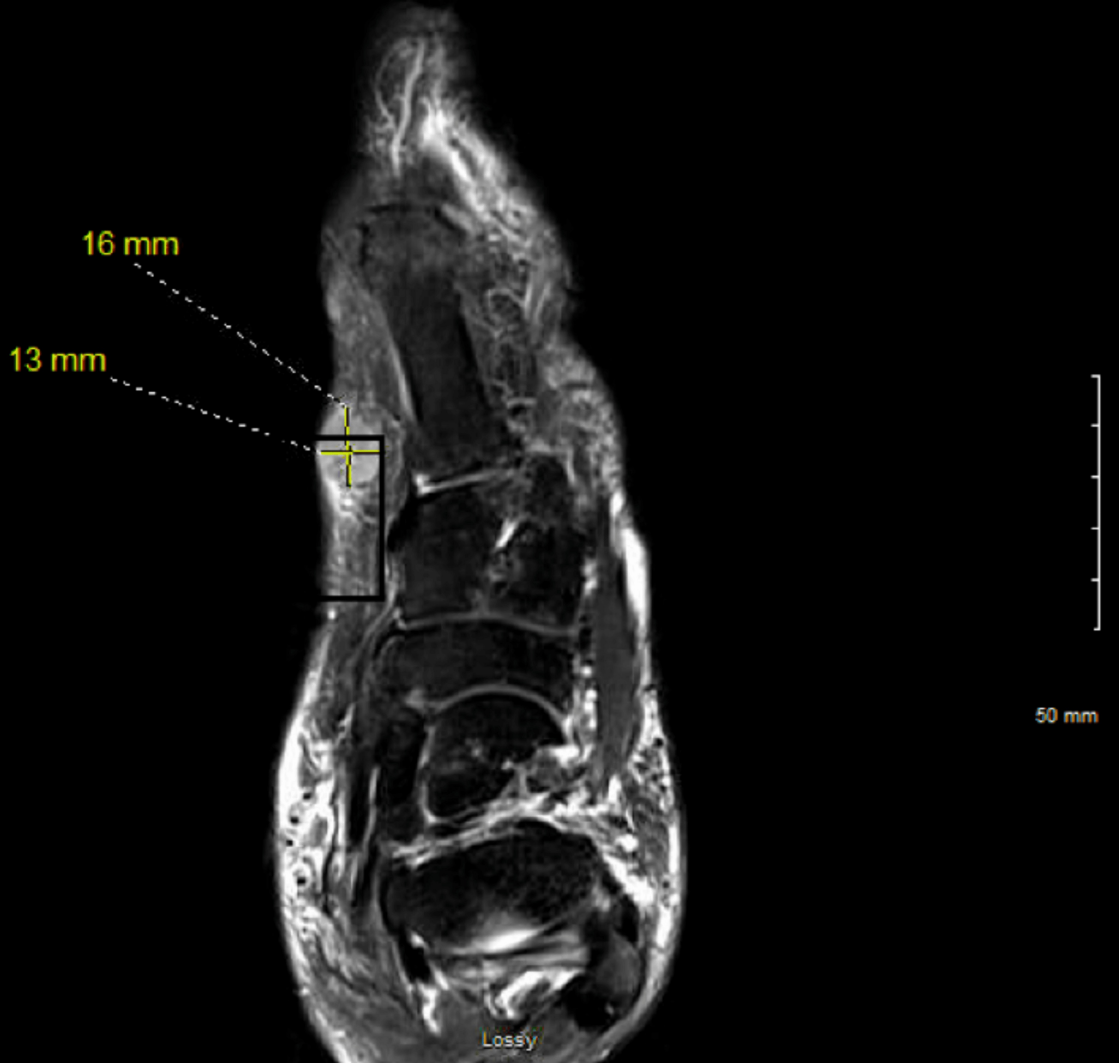
Left foot MRI (February 7, 2023) showing a 13 x 16 mm lesion on the plantar surface

The foot MRI was reviewed on February 7, 2023, which showed a lesion on the plantar left foot measuring 13 x 16 mm (Figure 1). A biopsy of the lesion was performed on February 9, 2023, by the podiatrist.

**Figure 2:**
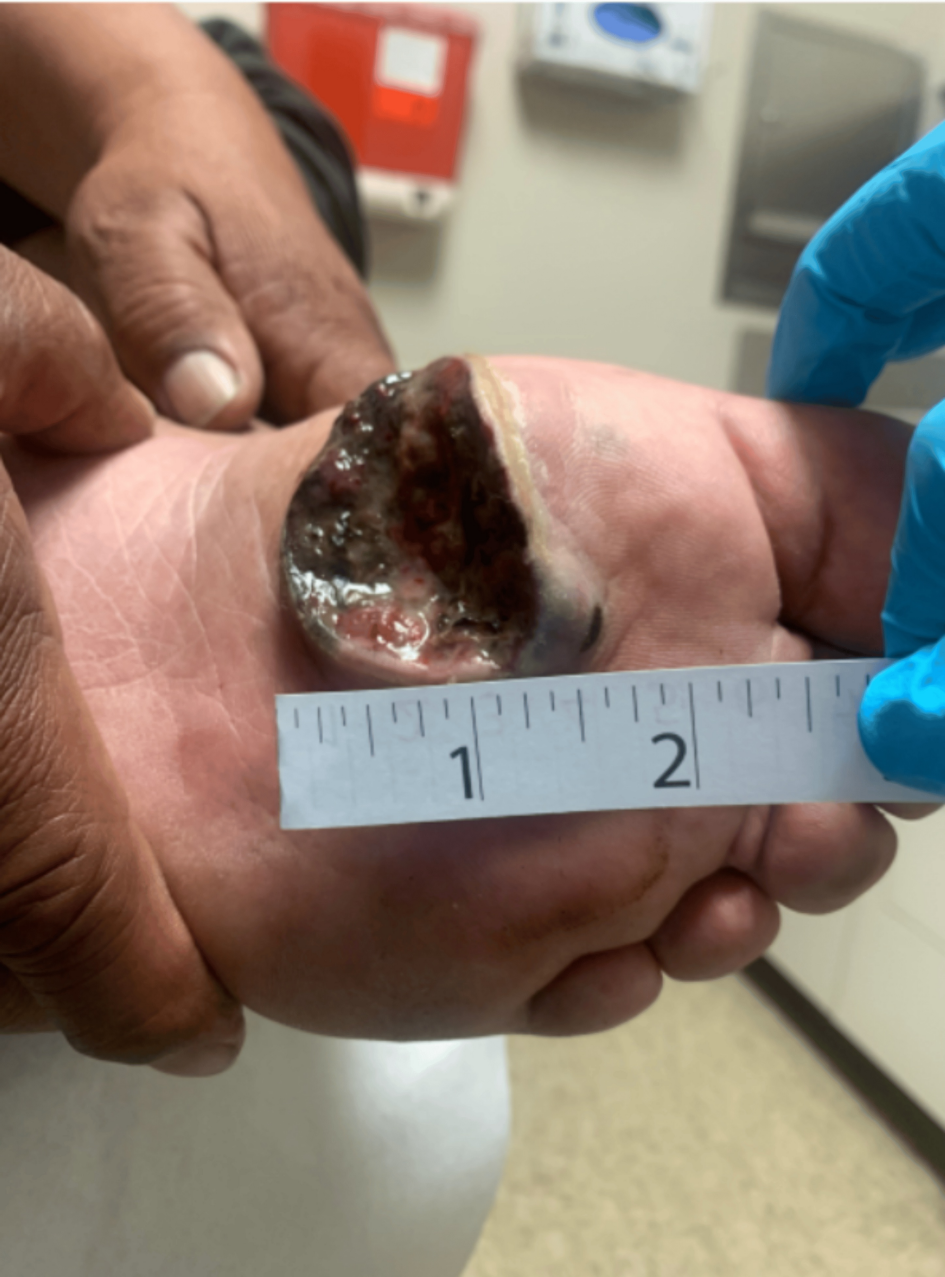
Plantar ulcer This figure shows a plantar ulcer with ill-defined borders, measuring 1.5 inches on the left foot. The image was taken by the physician on February 13, 2023.

**Figure 3:**
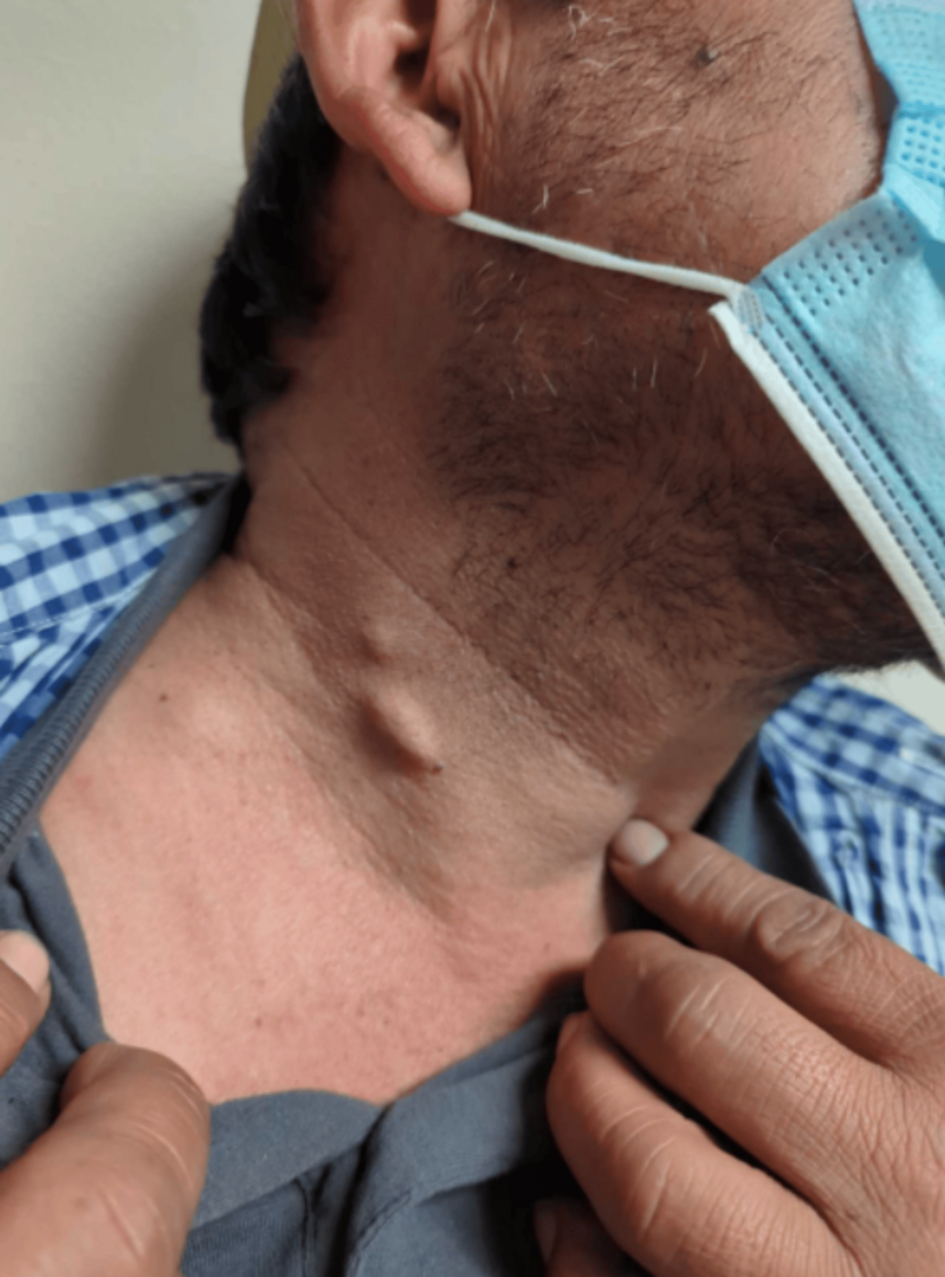
Swollen lymph nodes This figure shows two swollen anterior cervical lymph nodes. This image was taken by the physician on February 13, 2023.

**Figure 4:**
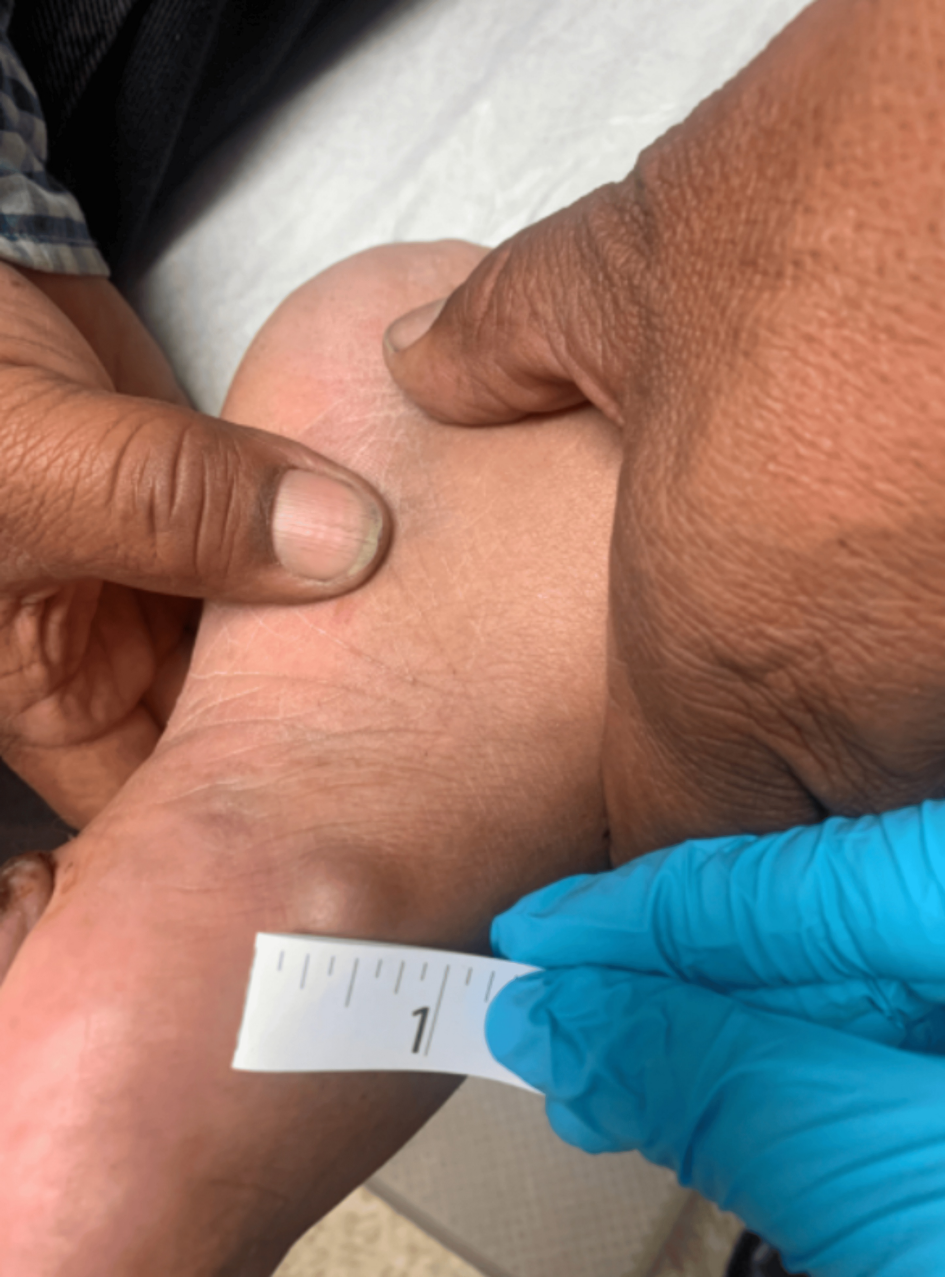
Foot nodule This figure shows a 1.5-inch nodule on the dorsomedial aspect of the left foot. This image was taken by the physician on February 13, 2023.

Then, on February 13, 2023, the patient was examined by the physician, who obtained photographs of his foot lesion (Figure 2) and other lesions on his body, including his torso, arm, neck (Figure 3), and dorsomedial surface of his left foot (Figure 4). The biopsy results, reviewed on February 15, 2023, confirmed a diagnosis of malignant melanoma of the foot, and the patient was referred to oncology. Ultrasounds were ordered for the multiple potentially metastatic lumps on his body. Later that month, the patient traveled to Mexico for a second opinion. There, a surgical oncologist removed his foot lesion and two lumps located on his right neck and left arm.

**Figure 5:**
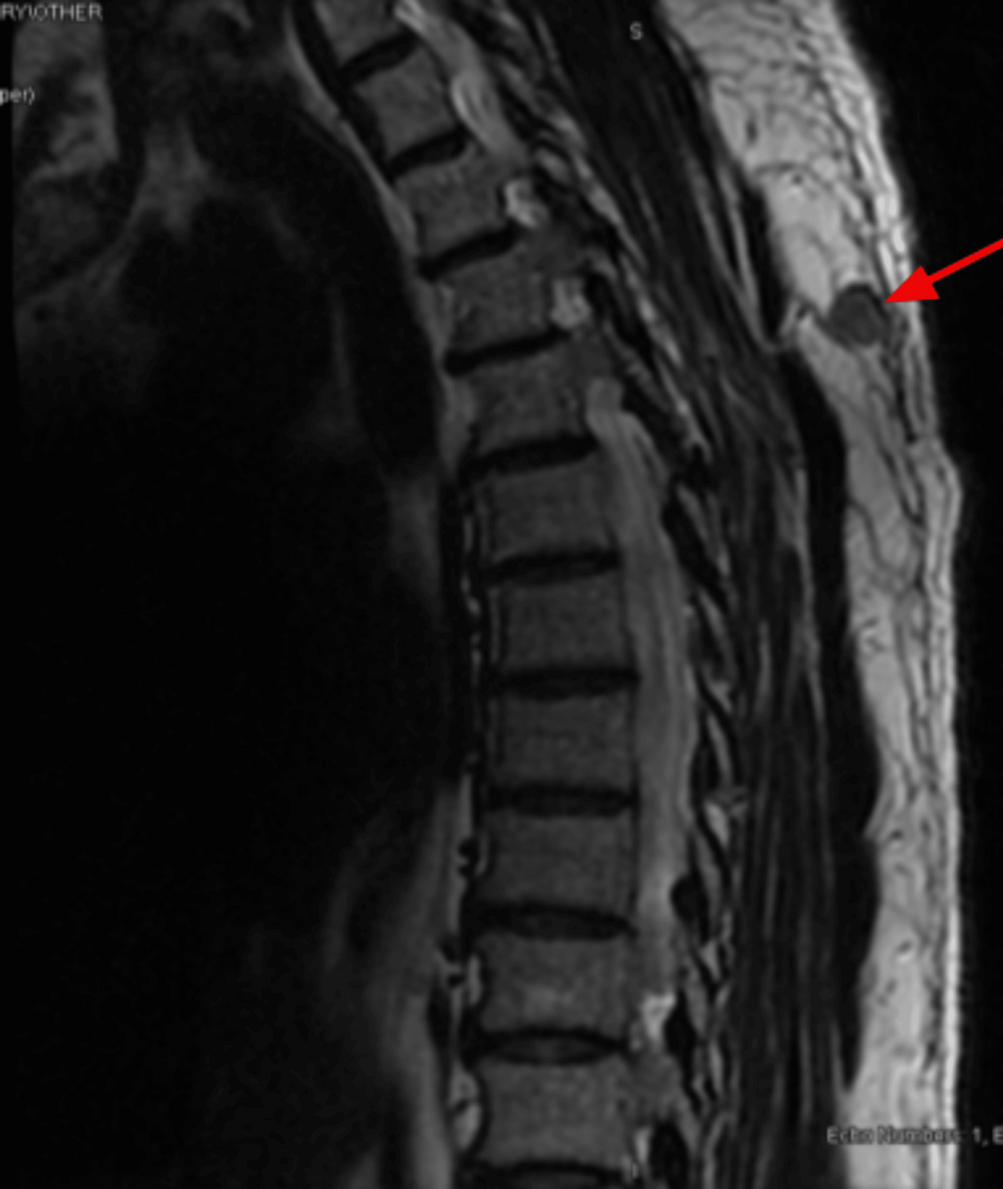
Spinal MRI (April 12, 2023) showing an enhancing hemorrhagic lesion in the soft tissue

**Figure 6:**
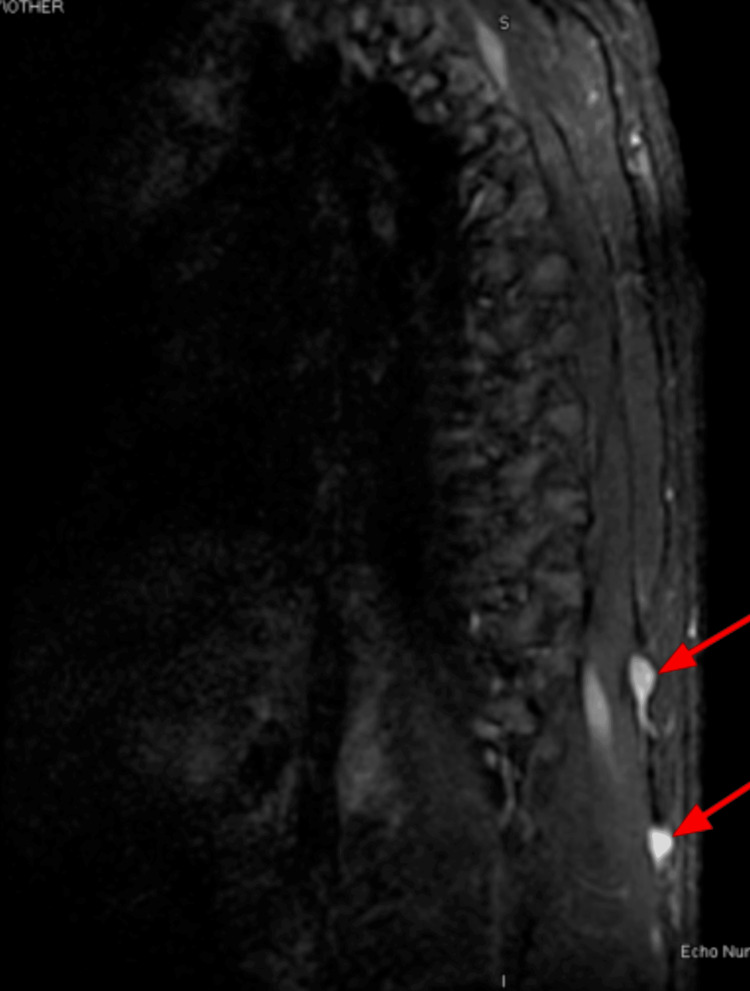
Spinal MRI (April 12, 2023) showing multiple enhancing hemorrhagic lesions in the soft tissue

**Figure 7:**
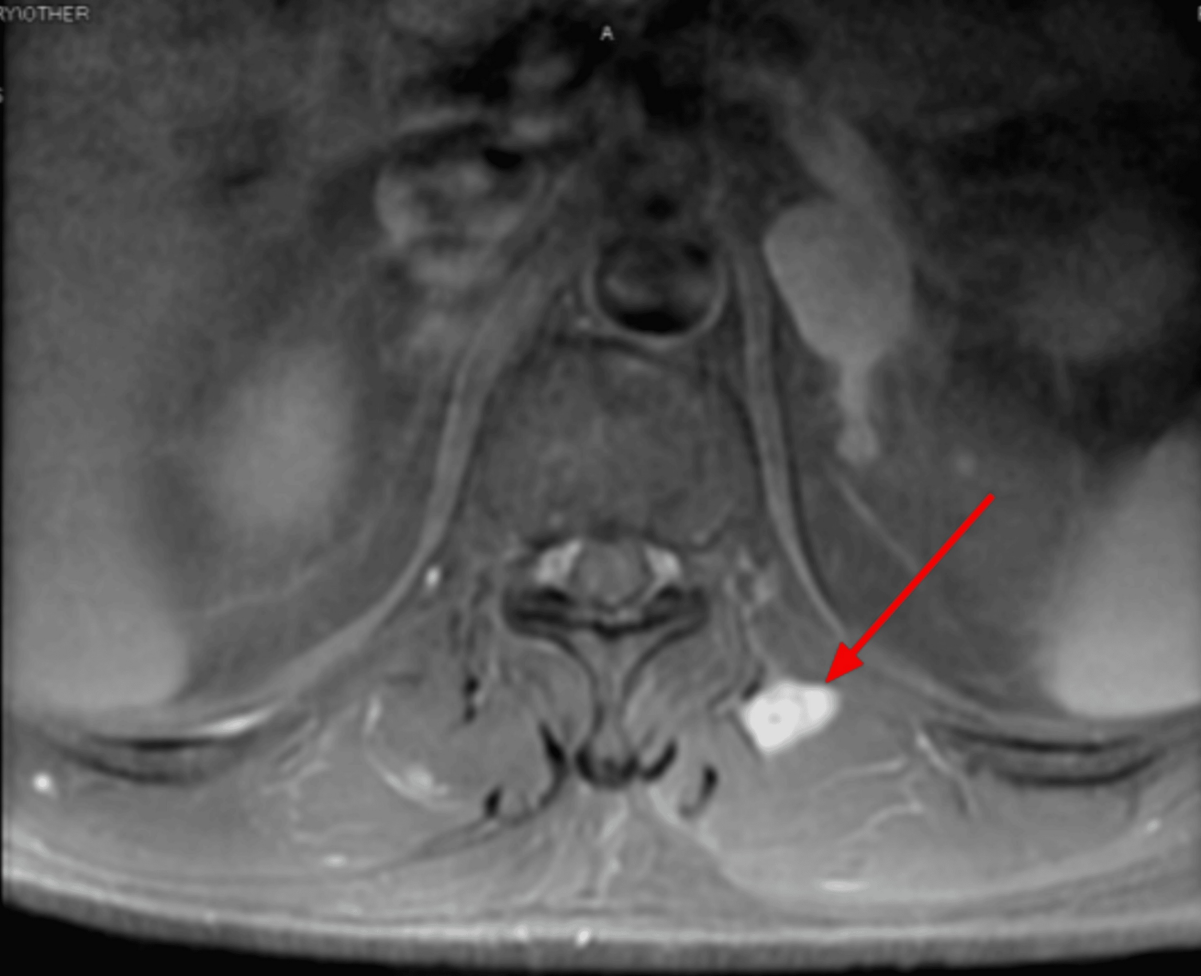
Chest CT (April 12, 2023) showing an enhancing hemorrhagic lesion in the soft tissue

**Figure 8:**
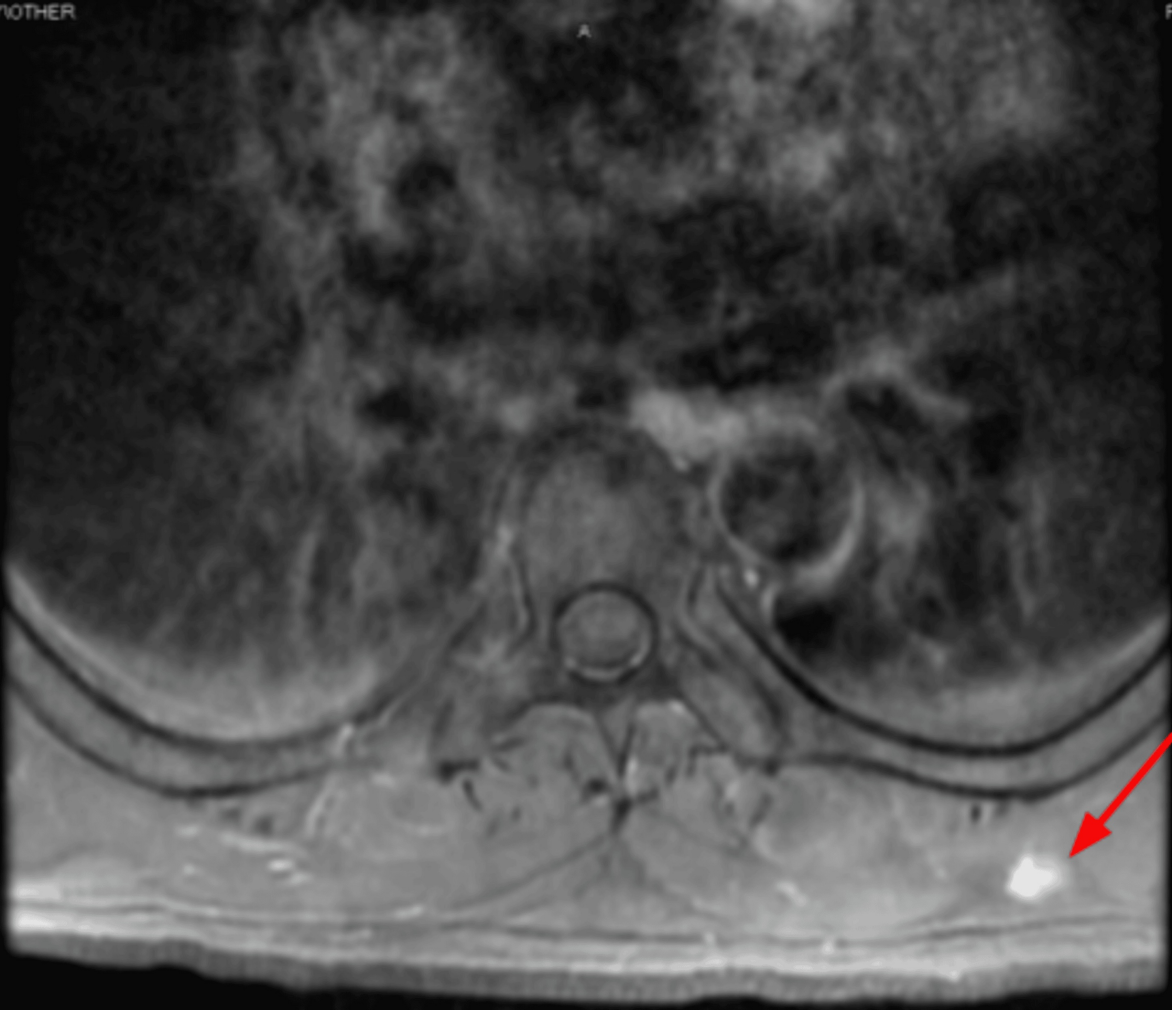
Chest CT (April 12, 2023) showing another enhancing hemorrhagic lesion in the soft tissue

In April 2023, the patient returned to the clinic to re-establish care. He was hospitalized for two days due to back pain, which his oncologist indicated was likely due to metastases. A spinal MRI (Figures 5, 6) and chest CT (Figures 7, 8) showed multiple enhancing hemorrhagic lesions in the soft tissues. Under the oncologist's management, the patient began chemotherapy and radiation therapy in the following months.

**Figure 9:**
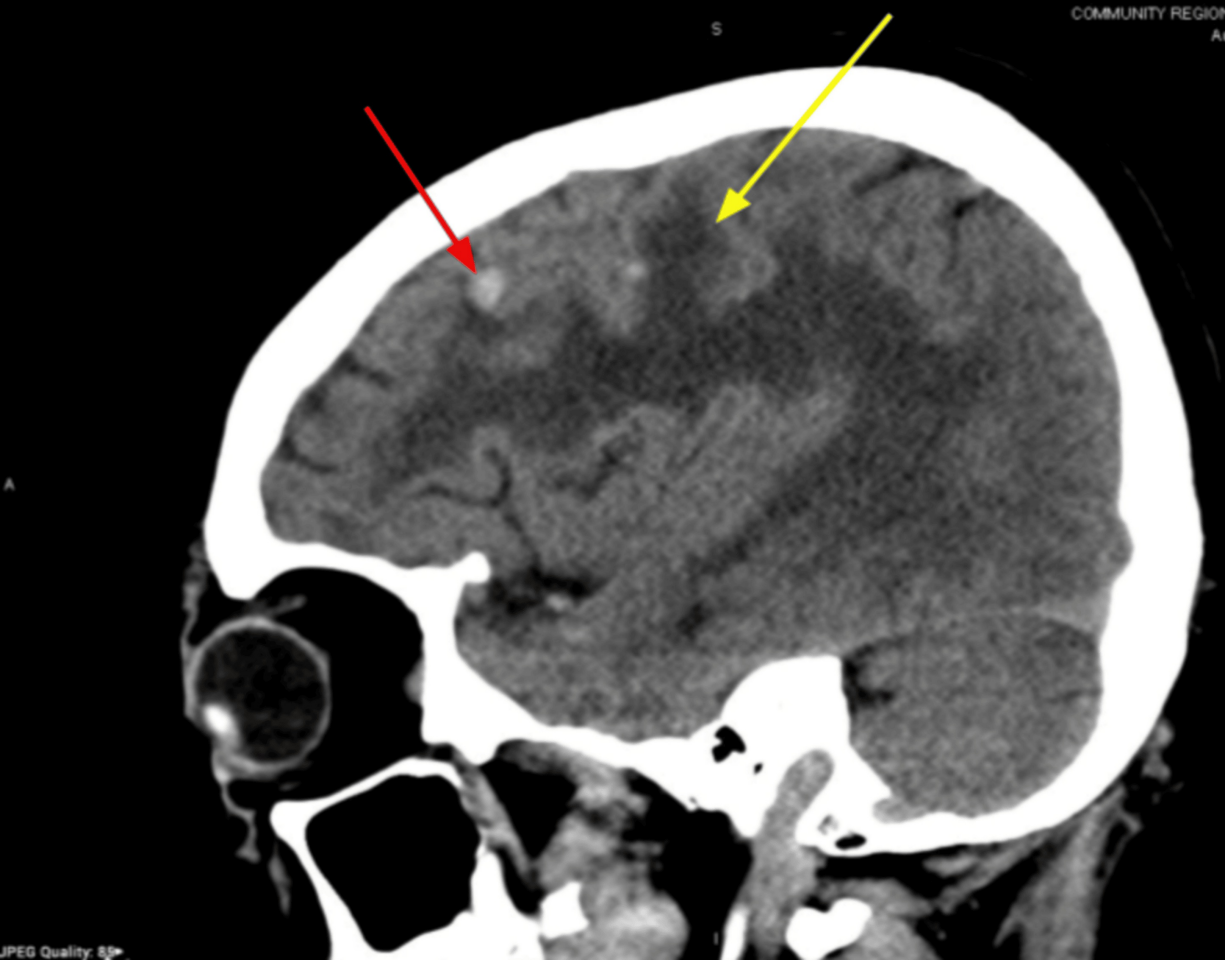
Brain MRI (October 2023) showing a left parasagittal frontal lobe lesion (red arrow) and surrounding vasogenic edema (yellow arrow)

Over the following six months, there was disease progression. In October 2023, an MRI showed a left parasagittal frontal lobe lesion with surrounding vasogenic edema (Figure 9).

**Figure 10:**
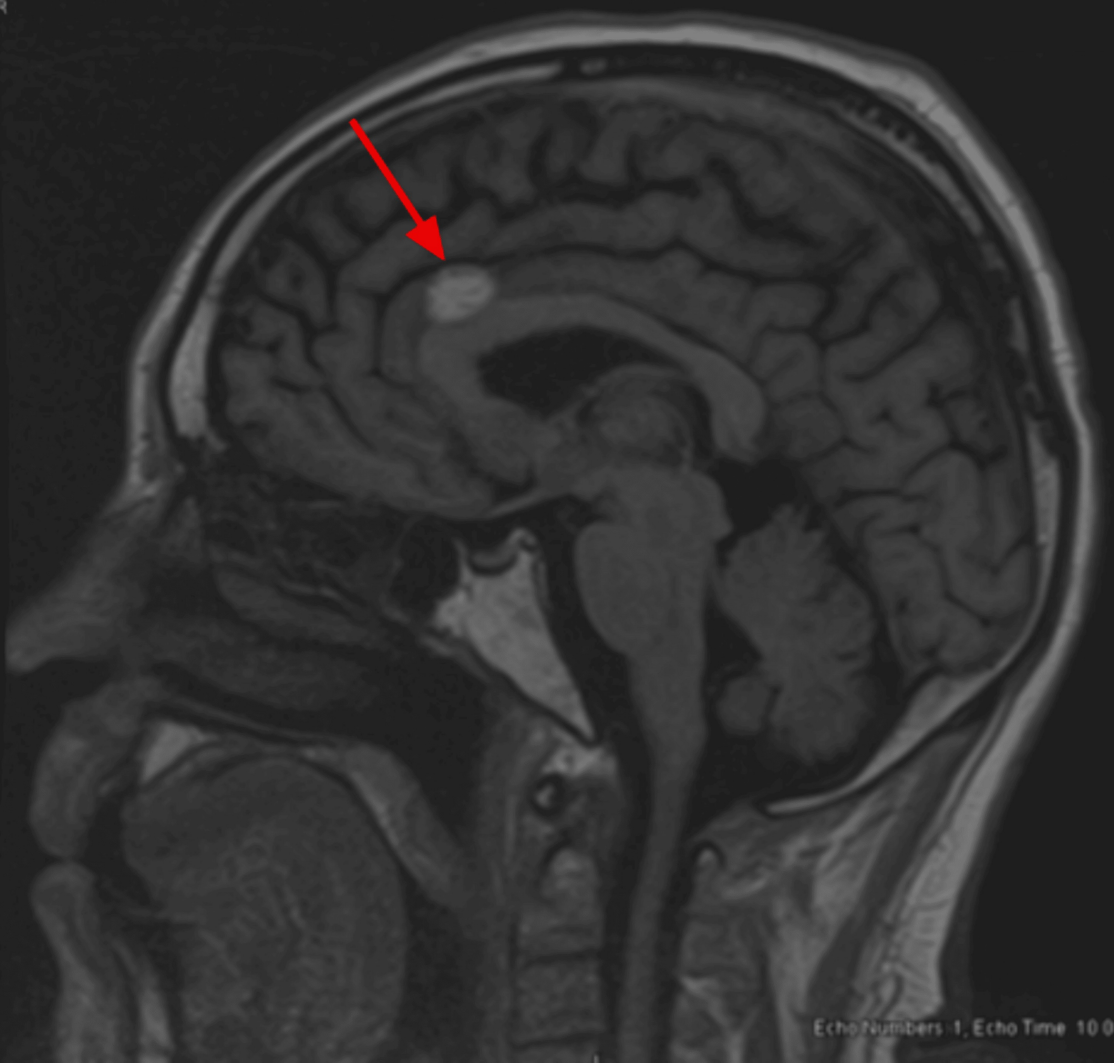
Brain MRI (May 2024) showing stability of metastases

**Figure 11:**
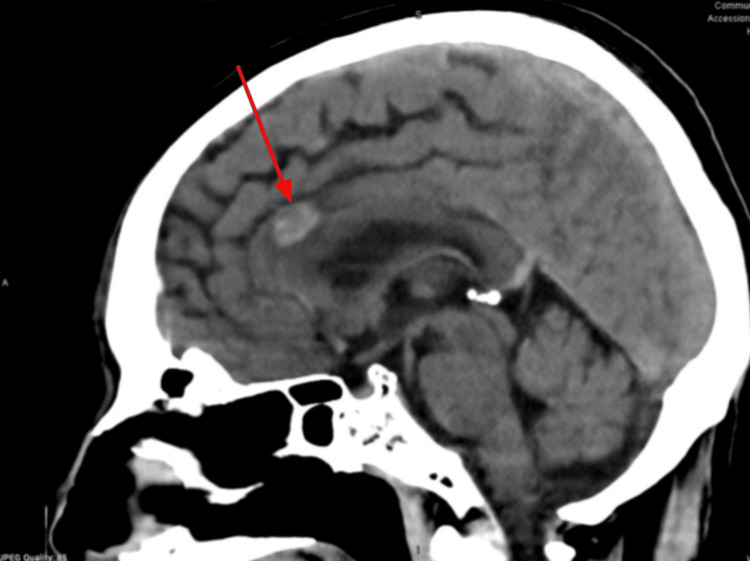
Brain MRI (June 2024) showing stability of metastases

**Figure 12:**
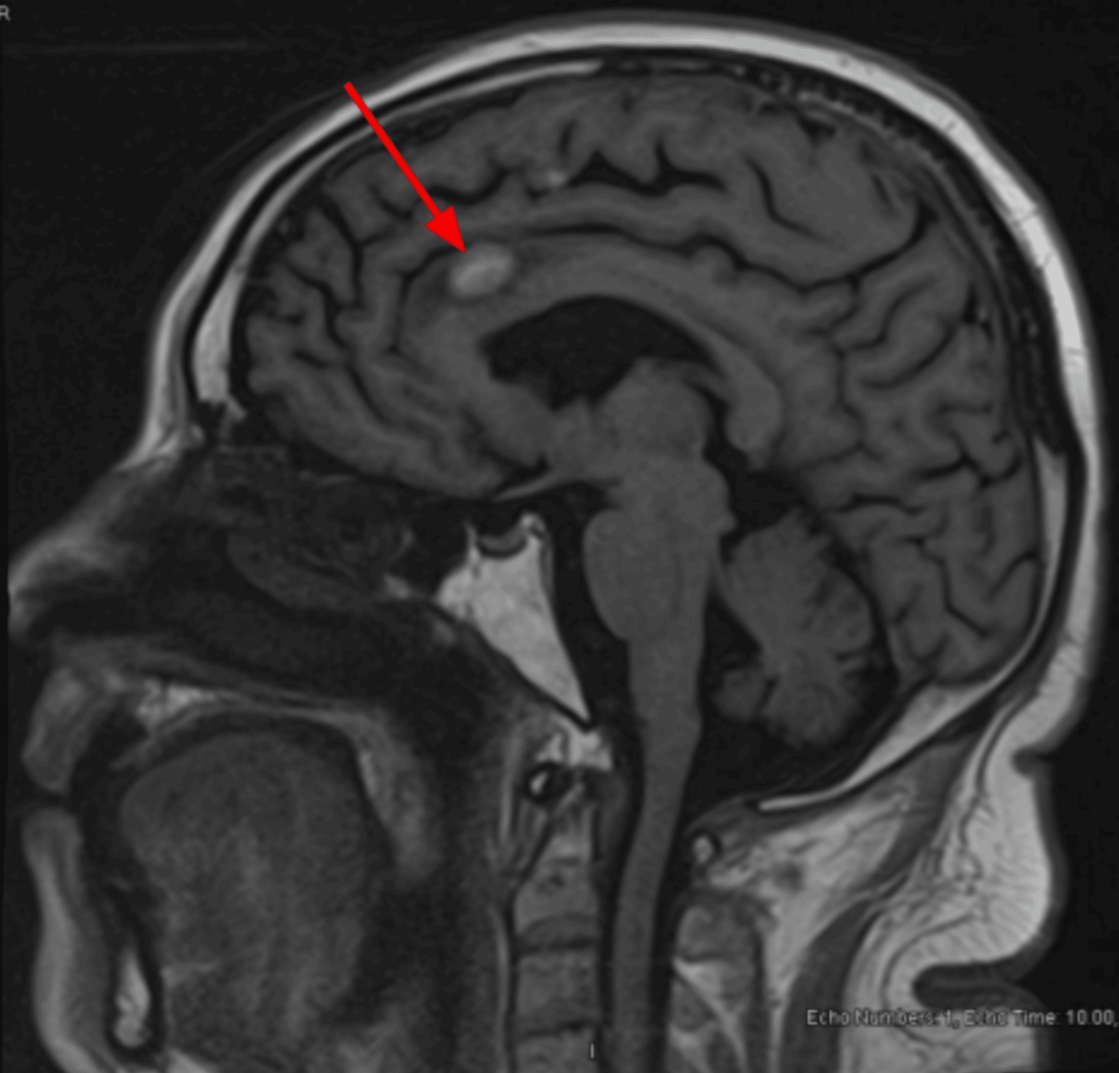
Brain MRI (September 2024) showing stability of metastases

**Figure 13:**
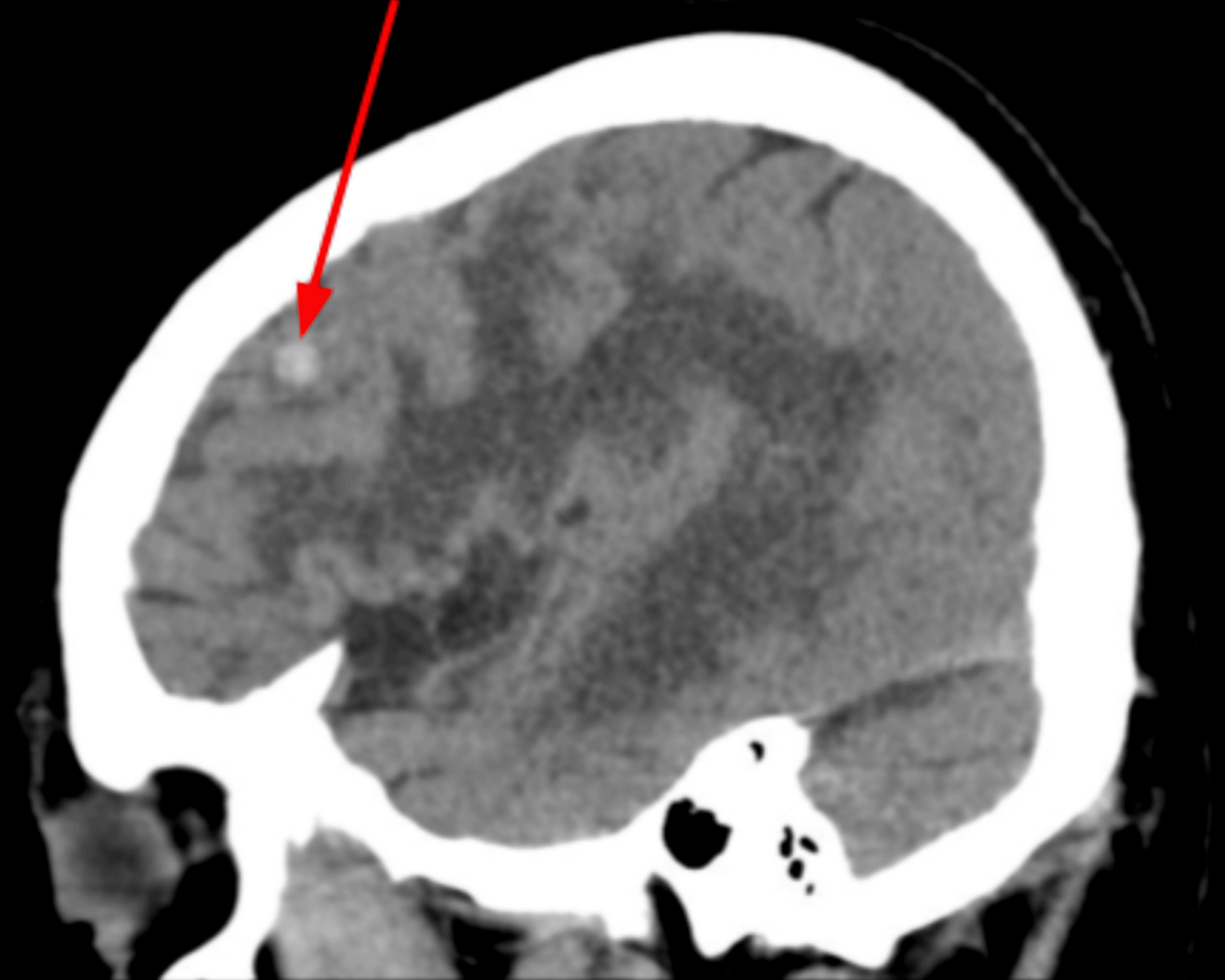
Brain MRI (October 2024) showing stability of metastases

Follow-up brain MRIs showed stability of the metastases in May 2024 (Figure 10), June 2024 (Figure 11), September 2024 (Figure 12), and October 2024 (Figure 13).

## Discussion

The presented case displays the challenges within healthcare systems, particularly in insurance access, communication, and trust. The patient experienced delays in care and struggled to grasp the severity of his condition due to a combination of language and cultural barriers. These barriers are symptomatic of broader socioeconomic disparities impacting healthcare access and quality. Due to the interwoven effects of social, economic, and institutional factors in shaping patient outcomes, efforts to address language barriers and enhance cultural competence among healthcare providers are pivotal for ensuring equitable care [8].

A noticeable feature of this case was the 10-week delay from the patient’s first hospital visit for his foot ulcer to the primary initiation of management, despite multiple attempts by the physician and the patient to resolve the insurance issue. Previous studies show that having continuous coverage makes a significant difference in whether patients can receive timely preventive care or recommended cancer screenings [7]. This case showcases the adverse effects of a lack of access to health insurance in regard to cancer progression. The situation faced by this patient is reflective of the barriers that chronically uninsured patients face when trying to maintain their health.

Language barriers hinder effective communication and lead to treatment delays [9]. In this patient's experience, limited communication hindered conveying the urgency of his condition, possibly influencing his decision to seek a second opinion in Mexico. Such instances reflect the intricate interaction between language proficiency and healthcare decision-making dynamics.

Moreover, the cultural factors of this case contributed to the delay in seeking medical attention for melanoma. The patient's prioritization of work responsibilities over health, a phenomenon documented by Reidy et al., underscores the impact of cultural norms on healthcare-seeking behavior [10]. It is common for patients of certain demographics such as that of the patient in this case (male, Hispanic, middle-aged) to value the characteristics of strength, resilience, and masculinity, often leading them to ignore seemingly minor physical inconveniences such as pain or skin deformities [11]. Furthermore, cancer is stigmatized in many cultures for its reputation of rendering patients ill and weak, and this fear often exacerbates delays in seeking care [12]. This cultural context, coupled with language barriers and insurance complexities, underscores the multifaceted challenges individuals face in navigating a healthcare system that often fails to consider their background.

Overall, various factors contributed to this patient’s cancer progression. With the delay in care, which started with the patient and was exacerbated by the medical system, the malignancy was able to develop unchecked and untreated. Deficiencies within the healthcare system to meet the needs of patients from traditionally underserved demographics played a role in hindering patient care, revealing room for institutional improvement.

## Conclusions

This case highlights the challenges associated with the delayed diagnosis and treatment of metastatic melanoma in underserved populations. Language barriers and cultural factors hindered effective communication between the patient and healthcare providers, delaying the initiation of appropriate diagnostic workup and subsequent management. A limitation of this report is the inability to attribute disease progression solely to treatment delays due to socioeconomic factors or to determine the exact degree of causality amidst other contributing factors such as natural disease progression. However, it is worthwhile to investigate the correlation between these social determinants of health and their outcomes to recognize the need for systemic improvements. Specific future recommendations include simplifying insurance processes for non-native English speakers and implementing high-quality translation services to effectively communicate with patients. Overall, efforts to improve health literacy, provide culturally sensitive care, and implement community-based screening programs are essential in reducing disparities in cancer outcomes.
